# Research on the Mechanical, Thermal and Induction Healing Properties of Asphalt Wearing Course with Steel Fibers

**DOI:** 10.3390/ma17092040

**Published:** 2024-04-26

**Authors:** Wei Liu, Shaopeng Wu, Quantao Liu, Jiazhu Wang, Pei Wan, Haiqin Xu, Qi Jiang

**Affiliations:** 1 State Key Laboratory of Silicate Materials for Architectures, Wuhan University of Technology, Wuhan 430070, China; liuw321@whut.edu.cn (W.L.); wanpei@whut.edu.cn (P.W.); xuhaiqin@whut.edu.cn (H.X.); jiang7001@whut.edu.cn (Q.J.); 2 Advanced Engineering Technology Research Institute of Zhongshan City, Wuhan University of Technology, Zhongshan 528400, China; 3 Fujian Provincial Transportation Research Institute Co., Ltd., Fuzhou 350004, China; wjzh1009@163.com

**Keywords:** asphalt wearing course, electromagnetic induction heating, temperature distribution, protective heating, self-healing

## Abstract

Induction healing technology can effectively repair microcracks in asphalt mixtures and is a promising maintenance technology for asphalt pavements. However, it requires the addition of steel wool fibers to asphalt mixtures and cannot be directly used to repair existing pavements. In order to improve the practicality of the induction healing technology, this article designs a wearing course asphalt mixture with induction healing function that is going to be paved above the existing road surface. The AC-10 asphalt wearing course for induction heating was prepared by adding steel fiber (SF). Analysis of the overall temperature of the surface revealed the unevenness of the temperature distribution, and the healing properties were investigated through protective heating that controlled the maximum temperature of the upper surface. The results show that the addition of SF can improve the high-temperature stability, low-temperature and intermediate-temperature crack resistance, and moisture stability of asphalt wearing courses; however, it has adverse effects on volumetric performance and skid resistance. The heating temperature increases with the increase in SF content, but higher maximum temperature heating rate causes worse heating uniformity and lower healing effect. The maximum heating rate of the sample with 10% SF reaches 3.92 °C/s, while its heating rate at minimum temperature is similar to that of the sample with 6% SF, which is only 0.7 °C/s, indicating the worst heating uniformity. The best healing effect occurs when the maximum temperature of the upper surface reaches 160 °C. The recommended optimal SF content is 6% of the asphalt volume. The asphalt mixture with 6% SF has an appropriate volume performance, moisture stability, and skid resistance; additionally, it has the best high-temperature stability, as well as low-temperature and intermediate-temperature crack resistance. Meanwhile, it also has uniform temperature distribution and efficient healing efficiency.

## 1. Introduction

In the field of pavement engineering, cracking is one of the main problems with asphalt pavements, mainly developing due to environmental effects and vehicle loads [1]. This shortens the service life of asphalt pavement, resulting in frequent maintenance and increased maintenance costs [2]. Research has found that asphalt is a viscoelastic material that is dependent on time and temperature [3]. Under longer time or higher temperature conditions, asphalt wets and merges at the crack edge through behavior similar to capillary flow, which then closes the cracks [3,4,5]. However, with increasing transportation and the high viscosity of asphalt during its service life, cracks propagate faster than self-healing at ambient temperatures [6]. Once microcracks develop into pavement macrostructural damage, relying on asphalt materials for self-repair will be meaningless [7]. To this end, researchers have conducted a lot of exploration to improve the durability and service life of the pavement.

In order to enhance the self-healing ability of asphalt pavement, the viscosity of asphalt must be reduced [6,8]. For this purpose, both healing agent-based and energy-based technologies have been developed. Encapsulated capsules release the healing agent under crack-tip stress, which not only reduces the viscosity of the surrounding asphalt but also reduces the aging of the asphalt [9,10,11]. However, controlling capsule rupturing is difficult, and the healing effect is one-time. Core-shell fibers can repair multiple cracks in multiple locations, but the healing effect is still one-time [12]. Energy-based healing techniques are more efficient and can be applied multiple times [13,14], mainly utilizing electromagnetic energy from different wavebands to increase the temperature of asphalt pavement. Among them, infrared heating mainly relies on infrared radiation to transfer energy to asphalt mixtures, but it takes a long time and consumes a high amount of energy [15,16]. Microwave heating uses microwave energy to cause material molecules to vibrate, thereby generating friction and heating the entire asphalt pavement [14,17]. Although efficient healing can be achieved, microwave radiation may cause thermal damage to the human body [18]. In addition, microwaves are usually reflected by flat surfaces and are difficult to control [3]. Electromagnetic induction heating technology can not only quickly heat the asphalt mixture and significantly improve its self-healing ability, but it also has the advantages of energy saving, environmental protection, safety, and high efficiency [19,20,21].

However, traditional asphalt concrete cannot be directly heated by induction energy, and conductive fillers (such as steel fiber [22,23,24], steel grit [25,26,27], steel slag [28,29], waste steel shaving [30,31], etc.) need to be added to enhance sensitivity to electromagnetic energy and induce thermal healing properties. At present, wearing course technology is a maintenance technology with obvious technical advantages. It extends the service life of the pavement by paving asphalt concrete with a thickness of 15~25 mm during its service life [32,33,34]. Therefore, adding steel fibers to a wearing course asphalt mixture can not only enhance the fracture toughness but also provide induced heat generation capabilities [20,21,35,36]. This approach effectively reduces the pollution and consumption of natural resources during the construction and production of asphalt pavement.

This paper aimed at studying the effect of steel fiber (SF) content on the road performance, thermal properties, and healing properties of induction-heated asphalt wearing course. Its volumetric performance and durability were comprehensively tested and compared with a control group without SF. To investigate the thermal properties of the wearing course, a FLIR infrared thermal imaging camera was used to monitor the heating process for 60 s and extract the temperature matrix. The non-uniformity of temperature distribution was revealed through the changes in the maximum, minimum, and average surface temperatures over time. Additionally, the relationship between the maximum temperature, average temperature, and heating time was established. Based on the thermal performance analysis results, a protective healing method was proposed to control the maximum temperature of the upper surface, effectively avoiding structural damage caused by local overheating. Finally, the healing performance was characterized by the fracture strength and fracture energy healing rates. This study can provide a reference for the application of induction heating technology in the wearing course.

## 2. Materials and Methods

### 2.1. Raw Materials

**Figure 1:**
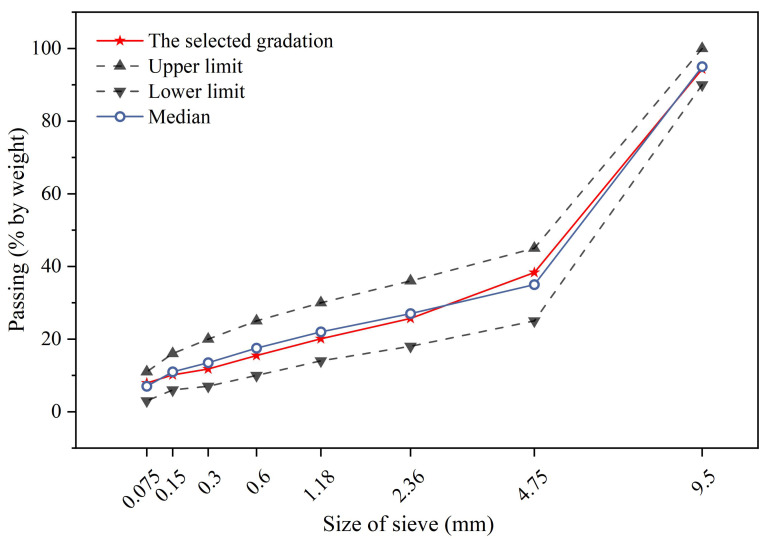
Gradation curve of AC-10.

The raw materials used in this study include high viscosity modified asphalt (HVA), basalt aggregate, limestone mineral powder, and SF. The properties of each material are listed in Table 1, Table 2 and Table 3, respectively. SFs were added in the form of admixtures according to the volume percentage of asphalt at 0%, 2%, 4%, 6%, 8%, and 10%, respectively, to prepare samples. The AC-10 grading curve is shown in Figure 1, and the optimal asphalt dosage was determined to be 5.7%. All of the materials meet the technical requirements of Technical Specification for Construction of Highway Asphalt Pavements (JTG F40-2004) [37].

### 2.2. Samples Preparation

**Figure 2:**
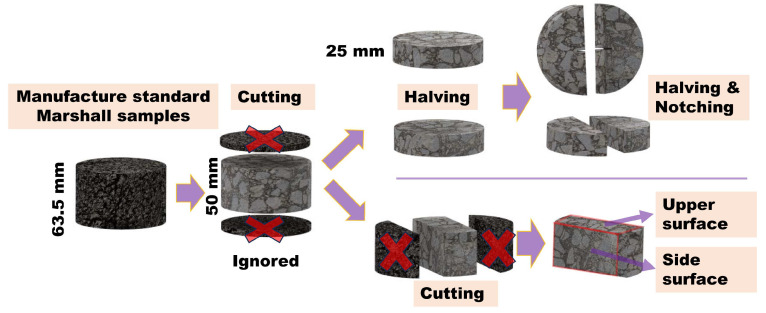
Schematic diagram of sample preparation steps.

The asphalt and aggregate were heated at 165 °C and 185 °C for 4 h, respectively, and then the asphalt mixture was mixed at 185 °C. A standard Marshall specimen was formed at 175 °C and compacted 75 times on both sides. The formed specimens were maintained at room temperature for 24 h. The sample preparation steps for the heating test and semi-circular bending (SCB) test specimens are shown in Figure 2. Finally, the dimensions of the SCB test sample were a semi-cylinder with a thickness of 25 mm, a diameter of 101.6 mm, and a notch with a height of 10 mm and a width of 1.5 mm. The temperature test sample is a block with a height of 50 mm, a length of 93 mm, and a width of 40 mm (as shown in Figure 2).

### 2.3. Experimental Method

#### 2.3.1. Volume Performance Test

First, the effect of SFs on the volumetric properties of the mixture was studied. The void volume (VV) and voids filled with asphalt (VFA) were tested according to ASTM D2726 [38].

#### 2.3.2. High Temperature Stability

This study utilized dynamic rutting tests to evaluate high-temperature stability performances. Dynamic stability (DS) refers to the number of rolling times required for each 1 mm deformation of the rutting slabs. DS reflects the high-temperature performance of asphalt mixture. The test was conducted in accordance with JTG F20-2011 [39]. Rutting slabs measuring 300 mm × 300 mm × 50 mm were prepared. Prior to the test, they were placed in a 60 °C constant temperature environmental chamber for 6 h. During the test, the rubber tire speed was set to 42 times/min and the tire pressure was 0.7 MPa. DS was calculated using Equation (1) [40].

(1) $$DS=\frac{\left(t_{2}-t_{1}\right)\times 42}{d_{2}-d_{1}}\times c_{1}\times c_{2}$$

where DS represents the dynamic stability of the asphalt mixture times/mm; t_1_ and t_2_ represent the test time, 45 min and 60 min, respectively; d_1_ and d_2_ represent the corresponding surface deformation of the test specimen at t_1_ and t_2_, respectively, mm; c_1_ is the testing machine-type coefficient, with a value of 1.0 when the loading wheel is in reciprocating operation mode; and c_2_ is the specimen coefficient, with a value of 1.0 when the specimen width is 300 mm.

#### 2.3.3. Low-Temperature Crack Resistance

This study employed the SCB test to investigate the low-temperature cracking resistance of induction-heated asphalt wearing courses with various SF contents. The test utilized the semicircular specimens outlined in Section 2.2. Forty samples were tested for each SF content. Prior to testing, the specimens were conditioned in a −10 °C environmental chamber for 6 h. The SCB fracture test was performed using UTM at a consistent loading rate of 0.5 mm/min, with the loading termination criterion being set to a load less than 0.5 kN.

#### 2.3.4. Intermediate-Temperature Crack Resistance

**Figure 3:**
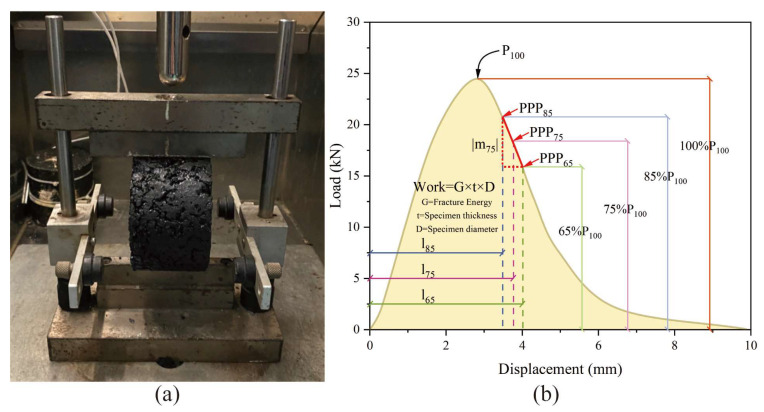
IDEAL-CT test: (**a**) test device and (**b**) typical load–displacement curve.

The intermediate-temperature crack resistance performance was evaluated following the standard test method ASTM D8225 [41]. Figure 3 depicts the test apparatus and a typical load–displacement curve of IDEAL-CT. A Marshall specimen with a diameter of 101.6 mm was directly utilized in IDEAL-CT. The test temperature was maintained at 10 °C, and the specimen was kept warm for at least 4 h. The UTM applied a loading rate of 50 mm/min, with the test stopping when the loading force fell below 0.1 kN. Four replicates were conducted for each mixture. Notably, |m_75_| represents the absolute value of the slope of the softening branch of the load–displacement curve at 75% of the maximum load, indicating the asphalt mixture’s crack propagation speed post-cracking. A smaller |m_75_| value suggests a greater capacity to resist crack extension. Its definition is outlined in Equation (2) [42]. The anti-cracking index CT_index_ provides a more accurate reflection of the asphalt mixture’s anti-cracking performance. A higher CT_index_ value signifies superior anti-cracking performance. Its definition is presented in Equation (3) [42].

(2) $$\left|m_{75}\right|=\left|\frac{P_{85}-P_{65}}{l_{85}-l_{65}}\right|$$

(3) $$CT_{index}=\frac{t}{62}\times \frac{G}{\left|m_{75}\right|}\times \frac{l_{75}}{D}$$

(4) $$G=\frac{W}{Dt}\times 10^{6}=\frac{\int Pdl}{Dt}\times 10^{6}$$

where D is the diameter of the specimen, mm; t is the thickness of the specimen, mm; m_75_ is the slope of the load–displacement curve at 75% of the peak force, kN·mm^−1^; P_85_ and P_65_ represent 85% and 65% of the peak load, respectively, kN; and l_85_, l_75_, and l_65_, respectively represent the displacement at 85%, 75%, and 65% of the peak load, mm.

#### 2.3.5. Moisture Stability

The relationship between the SF content and the moisture stability of asphalt mixtures was studied using the immersion Marshall test and the freeze–thaw splitting test. The immersion residual stability (MSR) refers to the ratio of the stability of a Marshall specimen immersed in 60 °C water for 48 h (MS_1_) to that after 30 min (MS). The freeze–thaw splitting strength ratio (TSR) refers to the residual splitting strength after the freeze–thaw process. MSR and TSR were calculated by Equations (5) and (6), respectively [43].

(5) $$MSR=\frac{MS_{1}}{MS}\times 100$$

(6) $$TSR=\frac{R_{T2}}{R_{T1}}\times 100$$

where R_T1_ represents the average splitting strength of specimens without freezing and thawing, MPa, and R_T2_ represents the average splitting strength of specimens after freeze–thaw cycles, MPa.

#### 2.3.6. Skid Resistance Performance

This study used the manual sand-laying method and the British Pendulum Number (BPN) test method to characterize the skid resistance of induction-type asphalt concrete. Both tests were carried out in accordance with JTG E60-2008 [44]. The calculation of BPN is shown in Equation (7), and the surface texture depth (TD) of the road is calculated using Equation (8) [45].

(7) $$BPN_{20}=BPN_{t}+\Delta BPN$$

where BPN_20_ indicates the swing value converted to a standard temperature of 20 °C; BPN_t_ is the swing value measured when the road surface temperature is t, where t = 25 °C; and ΔBPN is the temperature correction value, which is taken as 2 when the ambient temperature is 25 °C.

(8) $$TD=\frac{100\times V}{\pi \times D^{2}/4}=\frac{31831}{D^{2}}$$

where TD is the surface texture depth, mm; V is the volume of sand, 25 cm^3^; and D is the average diameter of the sand after flattening, mm.

#### 2.3.7. Induction Heating Test

**Figure 4:**
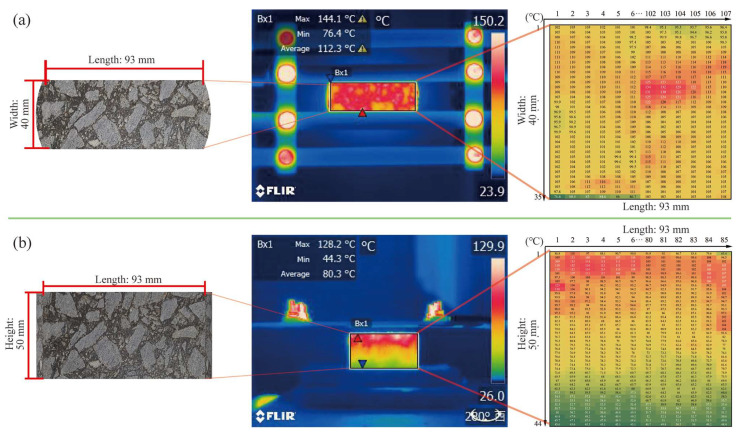
(**a**) Upper surface and (**b**) side surface temperature matrix extraction.

The parameters of the electromagnetic induction heating equipment (GH-IDDUCTION, EASYHEAT, GH Group in Valencia, Spain) were set to an output power of 9 kW, a frequency of 123 kHz, and a distance of 10 mm between the sample and the coil. An infrared camera with a resolution of 320 × 240 pixels (FLIR T420, Teledyne FLIR in Wilsonville, OR, USA) was used to monitor the changes in the sample surface temperature field. An infrared picture was taken every 10 s, and heating lasted for a total of 60 s. FLIR Tools software (version 6.0) was used to extract the sample surface temperature field data. As shown in Figure 4, the surface temperature of the specimen was quantitatively analyzed.

#### 2.3.8. Healing Performance Test

The fracture-healing test was conducted on SCB specimens with different SF content and involved the following four steps:

Step 1: The sample was placed at a temperature of −10 °C for more than 6 h; then the SCB specimen was tested once at a test temperature of −10 °C using UTM at a loading rate of 0.5 mm/min. Load to failure (load value ≤ 0.5 kN) and obtain the initial fracture strength F_1_ and initial fracture energy E_1_.

Step 2: Place the sample in a constant-temperature oven at 25 °C for 24 h to dry the sample.

Step 3: Conduct induction heating on the sample according to the system shown in Section 2.3.7. Before heating, lightly press both sides of the specimen to make crack contact. Control the upper surface temperatures of the specimen to be H1, H2, H3, H4, H5, and H6 (as shown in Section 3.8.1).

Step 4: Keep the sample in a constant-temperature oven at 25 °C for 24 h to regain strength.

Finally, repeat Step 1 to obtain the healed fracture strength F_2_ and the healed fracture energy E_2_.

The strength healing rate (HR_S_) is defined by Equation (9), and the fracture energy healing rate (HR_E_) is defined by Equation (13) [28].

(9) $$HR_{S}=\frac{F_{2}}{F_{1}}$$

(10) $$E=\frac{W_{f}}{A_{lig}}$$

(11) $$W_{f}=\int_{0}^{b}Fdu$$

(12) $$A_{lig}=\left(r-a\right)\times t$$

(13) $$HR_{E}=\frac{E_{2}}{E_{1}}$$

where F_1_ and E_1_ denote the initial critical load and fracture energy at the first fracture and F_2_ and E_2_ denote the critical load and fracture energy after healing. E denotes the fracture energy (J/m^2^), W_f_ denotes the work of fracture (J), A_lig_ denotes the area of the ligament (m^2^), and r, a, and t denote the radius of the sample (m), the notch length (m), and the thickness (m), respectively.

## 3. Results and Discussion

### 3.1. Influence of SF Contents on Volume Performance

**Figure 5:**
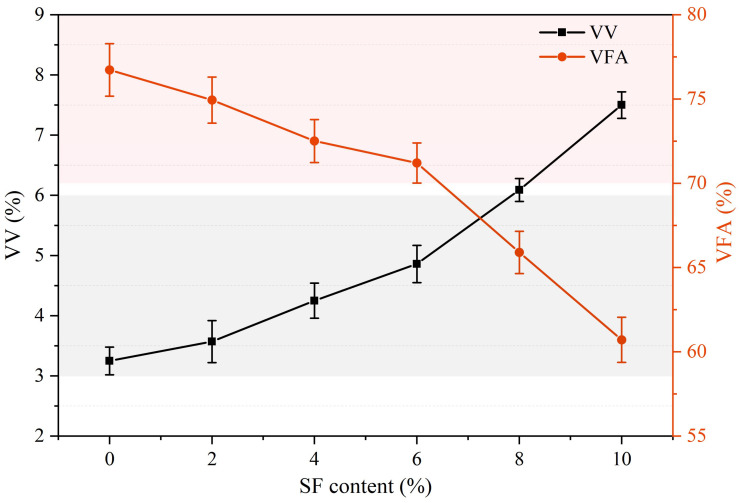
VV and VFA of asphalt wearing course with different SF contents.

The volumetric properties of samples with different SF contents are shown in Figure 5. It can be seen from the figure that, as the SF content increases, VV gradually increases and VFA gradually decreases. Specifically, the VV values of the test samples with SF content ranging from 0% to 10% were 3.25%, 3.57%, 4.25%, 4.86%, 6.09%, and 7.5%, respectively. The VV value range recommended by the specification is 3–6%. Obviously, when the SF content exceeds 6%, the VV value does not meet the specification requirements. In addition, the VFA values of the test samples with SF content ranging from 0% to 10% were 76.73%, 74.94%, 72.51%, 71.21%, 65.9%, and 60.71%, respectively. The VFA value range recommended by the specification is 70–85%. Obviously, the samples with 8% and 10% SF did not reach the lower limit required by the specification.

### 3.2. Influence of SF Contents on High Temperature Stability

**Figure 6:**
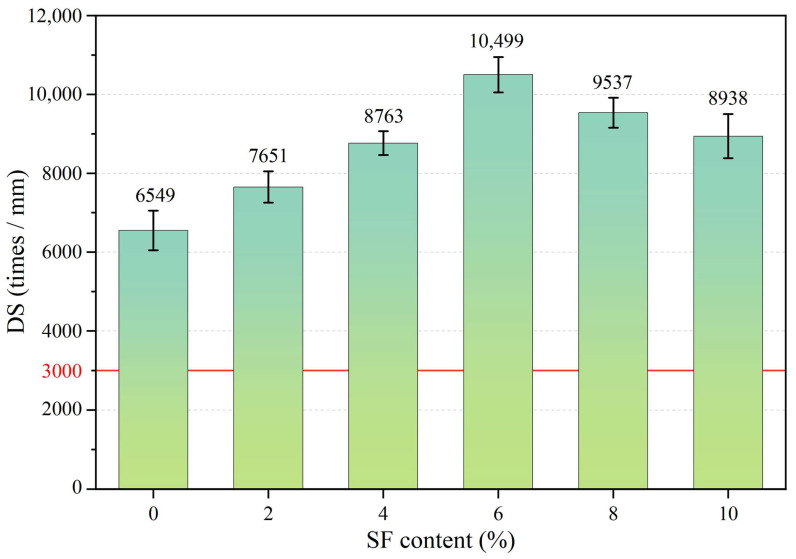
Dynamic stability of asphalt wearing course with different SF contents.

Figure 6 shows the dynamic stability test results of the sample. It can be seen that, as the SF content increases, DS first increases and then decreases. When the SF content is less than 8%, it is beneficial to increase DS, but the DS value of each sample is greater than 3000 times/mm. When the SF content reaches 6%, DS is the highest, reaching 10,499 times/mm. The increase in DS can be attributed to the following two points: on the one hand, SFs can increase the viscosity of asphalt materials; on the other hand, SFs form a spatial network structure in asphalt concrete that improves the ability of the mixture to resist plastic deformation. Based on Figure 5, it can be inferred that the reason for the decrease in DS may be that too many SFs can easily cause agglomeration, which affects the uniformity of the mixture and increases the VV. High VV leads to excessive gaps in mineral materials, loose skeleton structure, reduced internal friction, etc., which in turn leads to a decrease in thermal stability.

### 3.3. Influence of SF Contents on Low Temperature Crack Resistance

**Figure 7:**
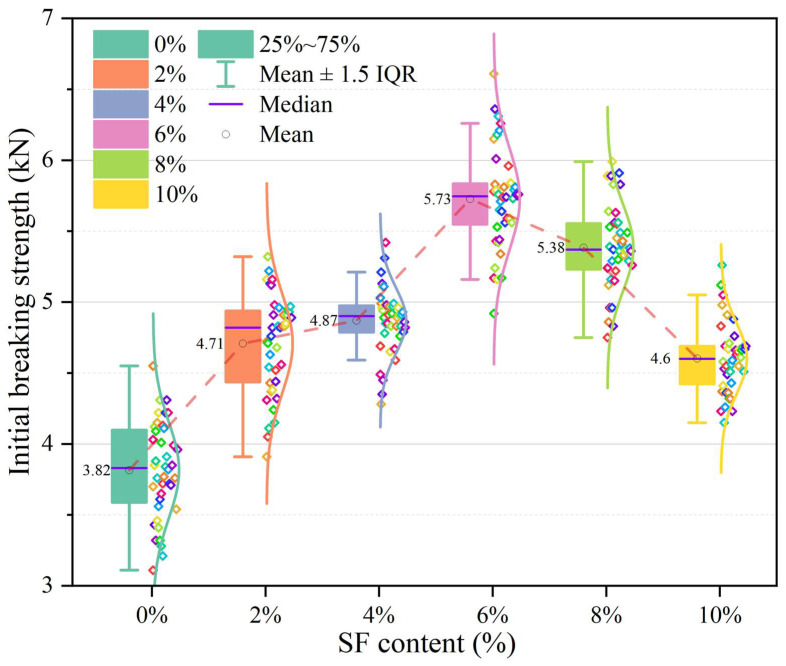
Initial fracture strength of asphalt wearing course with different SF content.

Figure 7 shows the relationship between the initial fracture strength and the SF content at low temperature. The colored dots in the figure represent the peak load of a single test specimen, while the numerical labels represent the average of 40 parallel specimens. The boxes represent the 50% value between the second and fourth quantiles of the data. The upper and lower edges of the error bars represent the distance of 1.5 times the IQR (interquartile range) of the data. Points exceeding this distance are considered outliers. The purple line segment in the box represents the median of the data, and the hollow circle represents the mean of the data. The curve to the right of the data points indicates that the data follows a normal distribution.

As shown in Figure 7, the test data is relatively concentrated and has few outliers. Therefore, it is reasonable to use the average fracture strength to analyze the test results. As the SF content increases, the initial fracture strength first increases and then decreases. Among them, the maximum initial breaking strength of 5.73 kN occurred when 6% SF was incorporated, while the original sample as a control group (that is, the SF content was 0%) showed the lowest initial breaking strength of 3.82 kN. In addition, it can be seen that the low-temperature crack resistance of 2% SF has been significantly improved (23% higher than the control group), while the sample groups with 2% SF (4.71 kN) and 4% SF (4.87 kN) showed similar low-temperature crack resistance. However, as the SF content continued to increase, its fracture performance was greatly enhanced. The sample with 6% SF is 0.86 kN higher than the sample with 4% SF, an increase of nearly 18%. However, as SFs continue to be added, their fracture performance begins to decline. The sample with 8% SF shows a fracture strength of 5.38 kN, while the fracture strength of the sample with 10% SF is only 4.6 kN, even lower than the sample with 2% SF.

The change in initial fracture strength may involve factors such as material strength, toughness, and the role of fibers. The following is a possible explanation: In the stage of increasing initial fracture strength, as the SF content increases, the SF plays a role in reinforcing the tensile strength of the material, thereby increasing the initial fracture strength. SF may also play a certain role in controlling crack expansion and preventing rapid expansion of cracks, thus improving the toughness of the material. During the initial fracture strength reduction stage, as the SF content continues to increase, aggregation effects between fibers may occur, resulting in an uneven distribution of SF in the concrete. This uneven distribution may result in reduced strength in some areas, leading to reduced initial fracture strength.

### 3.4. Influence of SF Contents on Intermediate Temperature Stability

**Figure 8:**
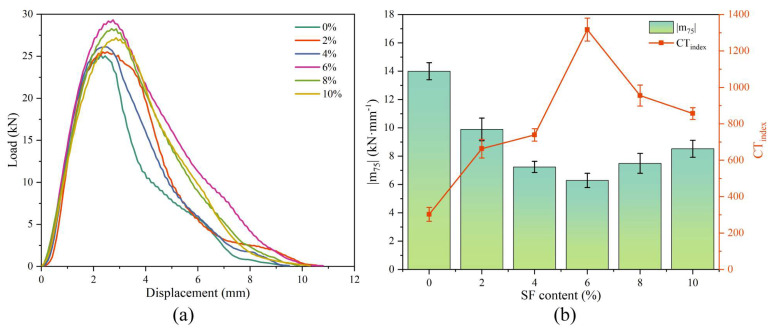
IDEAL-CT (**a**) load-displacement curve and (**b**) |m_75_| and CT_index_ calculation results.

Figure 8 shows the test results of IDEAL-CT. It can be seen from the figure that, as the SF content increases, the crack resistance of the mixture first increases and then decreases. The sample with 6% SF has the highest CT_index_ (1316.8) and the smallest |m_75_| (6.29 kN·mm^−1^). Compared with the control group, the CT_index_ increased by 435% and |m_75_| decreased by 44.9%. This is because the fibers enhance the crack resistance of the mixture and hold the specimen together as cracks propagate. In addition, samples with 4%, 6%, and 8% SF content have similar |m75| values; however, the CT_index_ values are significantly different, which is mainly attributed to the difference in fracture energy (as shown in Figure 8a). Furthermore, the crack resistance at intermediate temperatures follows a similar trend to that at low temperatures. Therefore, the explanation for the decrease in crack resistance at higher SF contents can also be attributed to the uneven distribution of fibers.

### 3.5. Influence of SF Contents on Moisture Stability

**Figure 9:**
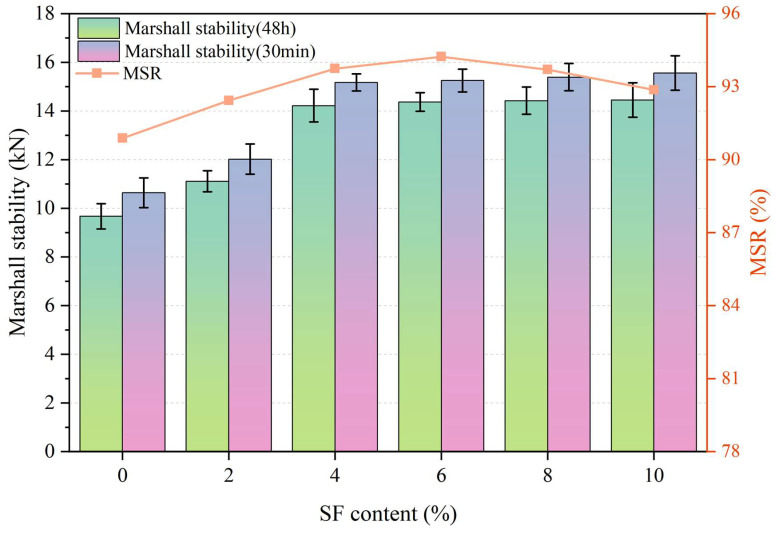
Marshall residual stability of asphalt course with different SF content.

**Figure 10:**
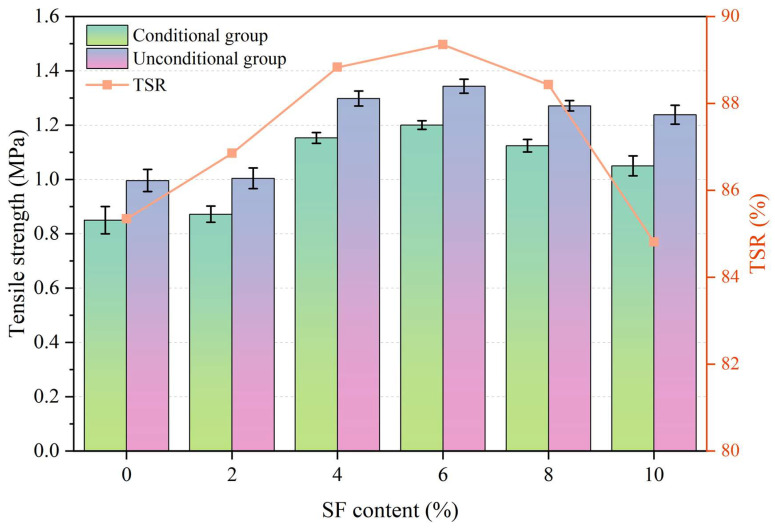
TSR of asphalt course with different SF content.

Figure 9 and Figure 10 show the moisture stability results of asphalt mixtures. As the SF content increases, the Marshall stability at different immersion times gradually increases, while the freeze–thaw splitting strength first increases and then decreases. The immersion Marshall test results of asphalt mixtures with different SF contents are shown in Figure 9. It can be seen from the figure that the residual stability of all asphalt mixtures is greater than 85%, which meets the technical requirements in the specification. With the addition of SF, the residual stability of the asphalt mixture shows a trend of first increasing and then decreasing. Compared to the asphalt mixture without SF, the residual stability of the asphalt mixture with 2% SF has the smallest improvement (92.43%), with an increase of only 1.7%. The 6% SF content has the best effect on improving the residual stability of the asphalt mixture, reaching 93.7%, an increase of 3.68%.

The freeze–thaw splitting test results of asphalt mixtures with different SF contents are shown in Figure 10. It can be seen from the figure that the splitting strength ratio of all asphalt mixtures far exceeds the 80% technical requirement in the specification. With the addition of SF, the splitting strength ratio of the asphalt mixture first increases and then decreases. Compared with the asphalt mixture without SF, the splitting strength ratio (84.81%) of the asphalt mixture with 10% SF was reduced by 0.62%, while the splitting strength ratio of the asphalt mixture with 6% SF was reduced by 0.62%. The splitting strength ratio had the best improvement effect, reaching 89.35%, an increase of 4.7%.

According to the above analysis, it can be seen that, as the SF increases, the moisture stability of the asphalt mixture gradually increases, reaching a peak at 6% SF content. However, a high SF content will also affect the moisture stability of the asphalt mixture. The splitting strength ratio and residual stability values may even be lower than those of asphalt mixtures with low SF content. The low content of SF mainly plays a reinforcing role in the asphalt mixture, which will make the bond between the asphalt mortar and the aggregate tighter. As the SF gradually increases to the optimal content, in addition to the reinforcing effect, the overlapping SF gradually forms a network structure, which helps the asphalt mixture resist moisture damage. When the SF content exceeds the optimal content, a certain degree of fiber agglomeration may occur. Partially agglomerated SF will lead to an increase in voids, weakening the asphalt mixture’s ability to resist moisture damage.

### 3.6. Influence of SF Contents on Skid Resistance Performance

**Figure 11:**
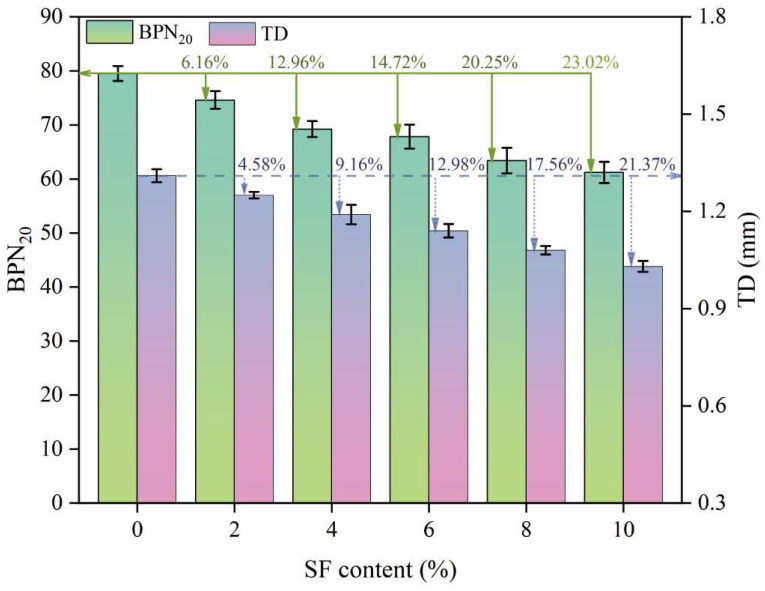
BPN and TD of asphalt wearing course with different SF contents.

The skid resistance performance test results are shown in Figure 11. It can be seen from Figure 11 that the incorporation of SF has a negative impact on the BPN, while the BPN continues to decrease as the SF content increases. The BPN of samples with SF content from 0% to 10% are 79.5, 74.6, 69.2, 67.8, 63.4, and 61.2, respectively, and the TD are 1.31 mm, 1.25 mm, 1.19 mm, 1.14 mm, 1.08 mm, and 1.03 mm, respectively. Compared with the sample with 0% SF content, as the SF content increases, the BPN decreases by 6.16%, 12.96%, 14.72%, 20.25%, and 23.02%, respectively. At the same time, the TD decreases by 4.58%, 9.16%, 12.98%, 17.56%, and 21.37%, respectively. It can be seen that the sample with 2% SF content has the least negative impact on the anti-skid performance. However, it is worth noting that the technical index requirements are BPN ≥ 45 and TD ≥ 0.6 mm, respectively. Therefore, the BPN and TD of all test samples meet the technical index requirements. The decrease in anti-skid performance can be attributed to the increased coating of asphalt due to the overlap of SF between aggregate particles, resulting in a smoother surface, reduced structural depth, and a lower friction coefficient.

### 3.7. Influence of SF Contents on Thermal Performance

#### 3.7.1. Induction Heating Efficiency

**Figure 12:**
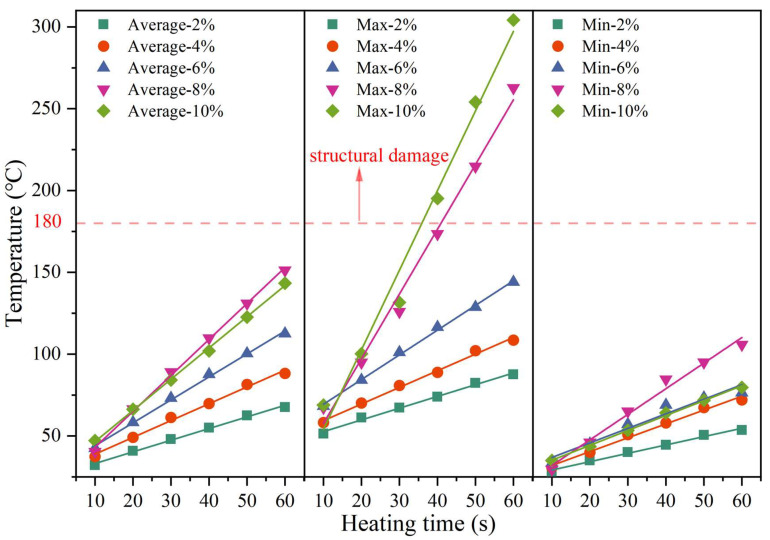
Changes in heating temperature with time for samples with different SF content.

**Figure 13:**
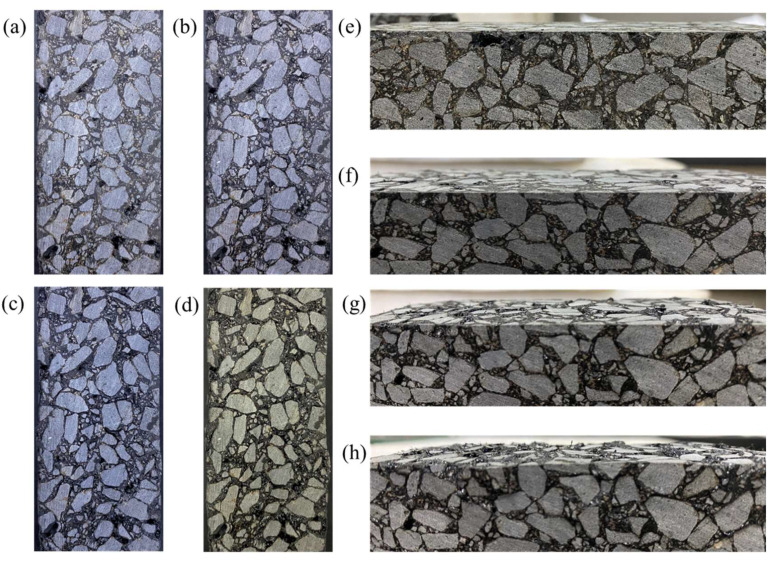
The surface morphology of the sample containing 6 %SF when the upper surface temperature reaches (**a**,**e**) unheated, 80 °C, 100 °C, 120 °C (**b**,**f**) 140 °C (**c**,**g**) 160 °C and (**d**,**h**) 180 °C.

**Figure 14:**
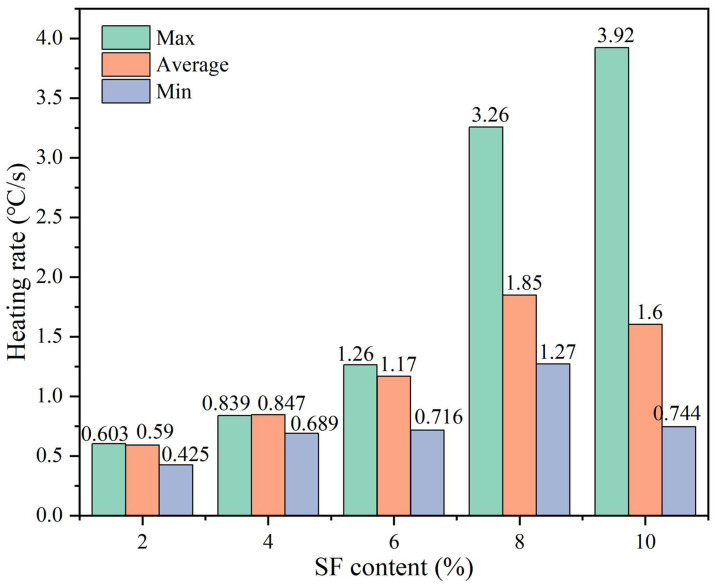
Heating rates at the maximum, minimum and average temperatures for samples with different SF content.

Figure 12 shows the changes in the maximum, average, and minimum temperatures of the upper surface with heating time and SF content. The linear fitting results are shown in Table 4. The R^2^ values are all greater than 0.98, indicating that temperature and time are highly linearly related. It can be seen from Figure 12 that, as the SF content increases, the surface temperature gradually increases. However, the average temperature, maximum temperature, and minimum temperature of the samples with 8% and 10% SF showed different changing trends. When heated for 60 s, the maximum temperature of the sample with 10% SF (304 °C) was 41 °C higher than the maximum temperature of the sample with 8% SF (263 °C). The lowest temperature of the sample with 10% SF (80 °C) is similar to the lowest temperature of the sample with 6% SF (76 °C), which is 26 °C lower than the lowest temperature of the sample with 8% SF (106 °C). This reflects the uneven distribution of temperature under induction heating and results in the average temperature of the sample with 8% SF (151 °C) being higher than the average temperature of the sample with 10% SF (143 °C). In addition, during the test, it was found that, when the maximum temperature of the upper surface reached 180 °C, the mixture began to suffer local structural damage (as shown in Figure 13). Therefore, the maximum temperature of the upper surface needed to be controlled below 180 °C in subsequent healing tests. It can be seen from Figure 14 that the heating rate at the maximum temperature increases with the increase in the SF content, while the heating rates at the minimum temperature and the average temperature show a trend of first increasing and then decreasing with the increase in the SF content, with the peak values appearing at 8% SF content. The heating rate at the maximum temperature of the sample with 10% SF content reached 3.92 °C/s, while its heating rate at the minimum temperature was similar to that of the sample with 6% SF, which was only about 0.7 °C/s, indicating the worst heating uniformity.

#### 3.7.2. Effective Heating Depth

**Figure 15:**
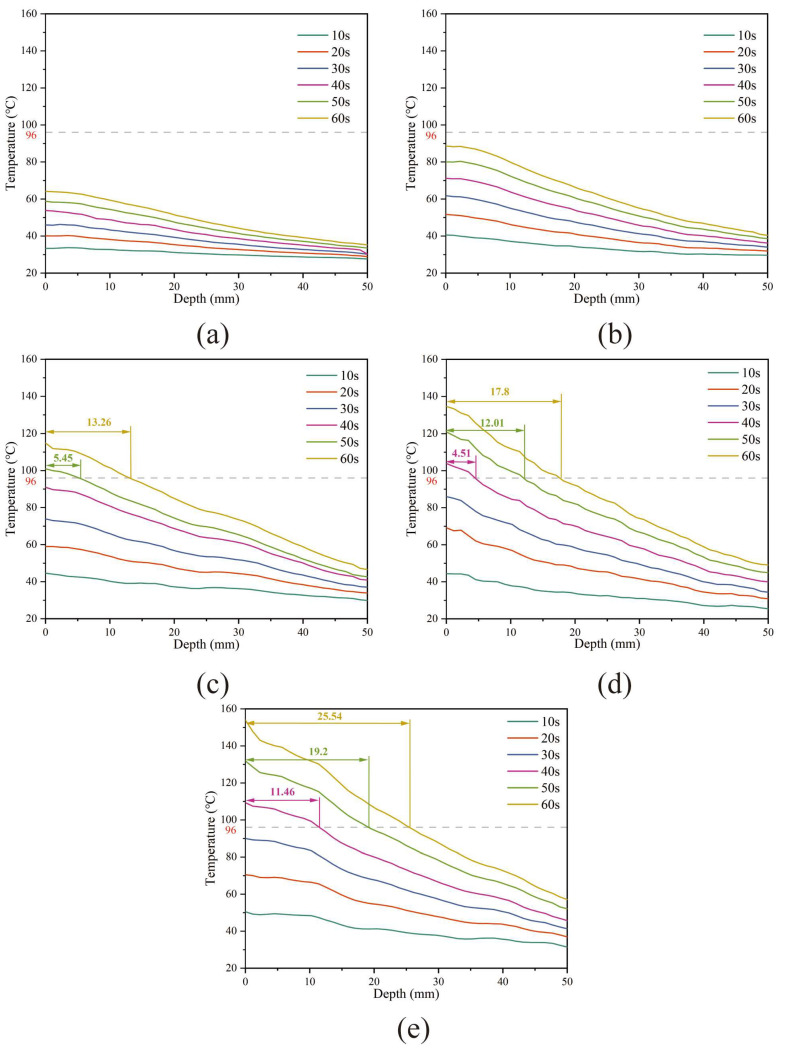
EHD of samples with (**a**) 2%, (**b**) 4%, (**c**) 6%, (**d**) 8%, and (**e**) 10% SF content.

The effective heating depth (EHD) is defined as the maximum depth at which the average temperature reaches the softening point temperature. According to the temperature matrix extraction method shown in Figure 4 (see Section 2.3.7), the side surface temperature matrix row numbers are converted into actual depth according to Equation (14). As shown in Figure 15, the gray dashed line in the figure represents the softening point temperature (96 °C) of the HVA used in this study, while the distance marked in the figure indicates the actual EHD. It is obvious that, within the test time of 60 s, the samples with 2% (Figure 15a) and 4% (Figure 15b) SF do not have EHD. When the SF content continues to increase, the EHD increases with the increase in SF. Taking heating for 60 s as an example, the EHD for SF content of 6%, 8%, and 10% is 13.26 mm, 17.8 mm, and 25.54 mm, respectively. Additionally, the time for the EHD to appear gradually shortens; for example, when the SF content is 6%, the EHD is first calculated after heating for 50 s, while, when the SF content is 8%, the EHD is calculated after heating for 40 s. Although the EHD gradually increases with the SF content, the curve of temperature with depth also becomes steeper, indicating that the temperature difference in the depth direction gradually increases and the temperature uniformity worsens. The temperature of low-SF content samples changes relatively slowly with depth. When the heating time is extended, the overall temperature can be increased more uniformly. This also explains the phenomenon that the sample with 2% SF in Section 3.8.2 has the highest healing rate.

(14) $$H_{1}=H_{2}/H_{1}\times C_{i}$$

where H_1_ is the actual height value, 50 mm; H_2_ is the number of temperature matrix rows, 44; and C_i_ is the temperature matrix row number, i = 1, 2, 3, …, 44.

### 3.8. Influence of SF Contents on Healing Performance

#### 3.8.1. Determine Heating Temperature

**Figure 16:**
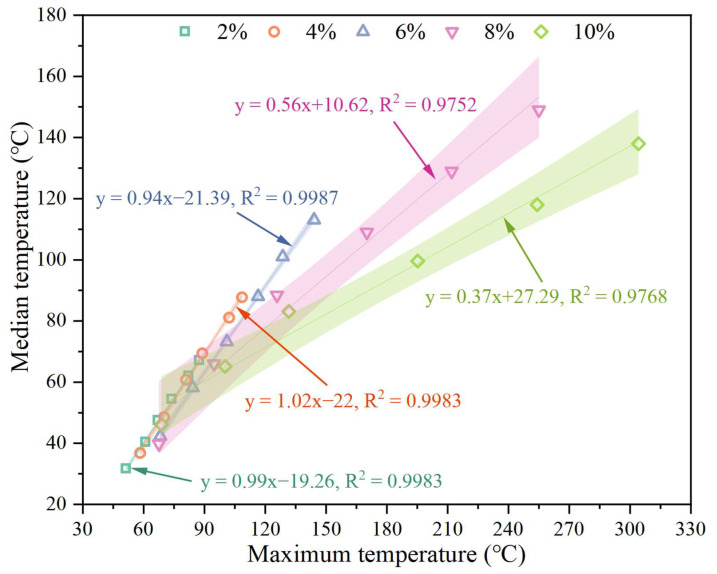
Linear fitting results of upper surface average and maximum temperature.

Based on the induction heating performance test results, this study adopts a heating method that controls the maximum temperature of the upper surface and stops heating immediately when the maximum surface temperature reaches the set value. As shown in Figure 16, according to the analysis results in Section 3.7.1, the linear relationship between the maximum temperature and the average temperature is constructed through linear fitting. The fitting parameters are shown in Table 5. The fitting goodness is greater than 0.97 under each SF content, indicating that the fitting results have a good linear relationship. Since the softening point temperature of HVA is 96 °C, actual tests found that when the maximum temperature of the upper surface exceeds 180 °C, the overall structure of the asphalt concrete begins to lose stability. Therefore, the maximum temperatures of the upper surface are controlled as follows: H1 (no heating), H2 (100 °C), H3 (120 °C), H4 (140 °C), H5 (160 °C), and H6 (180 °C). Based on the linear fitting results in Table 4 and Figure 16, the average temperature and heating time corresponding to the control temperature under each SF content were calculated. The specific results are listed in Table 5.

#### 3.8.2. Healing Effect Analysis

**Figure 17:**
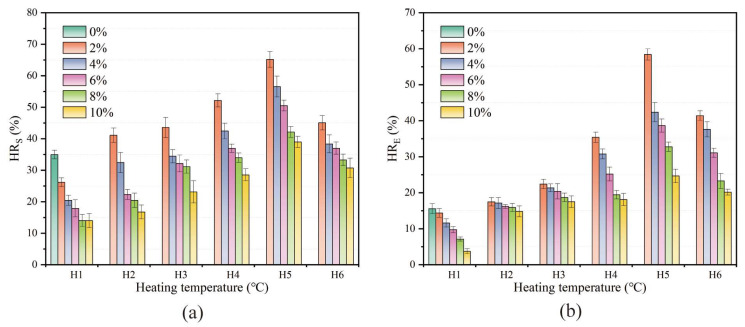
(**a**) Fracture strength healing rate and (**b**) fracture energy healing rate of samples with different SF content at different upper surface maximum temperatures.

The healing properties were studied according to the steps described in Section 2.3.8. The healing properties of samples with different SF at different heating temperatures were analyzed. The control sample with 0% SF cannot be heated by induction, so it only healed at room temperature (25 °C). The samples with other contents were heated according to the maximum temperature of the upper surface shown in Table 5. As shown in Figure 17, the healing effect was evaluated through two indicators: the fracture strength healing rate (HR_S_) and fracture energy healing rate (HR_E_). The results show that, at the same heating temperature, as the SF content increases, the healing rate gradually decreases. For samples with the same SF content, as the heating temperature increases, the healing rate first increases and then decreases, showing a consistent trend among samples with different SF content. The samples with 2% SF showed the best healing effect at H5. At this time, the HR_S_ was 65.15% and the HR_E_ was 58.4%. This is because the heating uniformity is different with different SF contents. For the sample with 2% SF, it takes the longest time (160 s) to reach the control temperature of H5, the temperature distribution is the most uniform, the overall temperature can be increased simultaneously, and more asphalt flows or expands to heal the crack. At heating temperatures from H2 to H5, the healing effect is always the best when the SF content is 2%, and the optimal healing temperature is H5. This may be due to two reasons: On the one hand, the initial fracture strength of the sample with less SF content is lower (as shown in Figure 7); on the other hand, the sample with less SF content took longer to reach the same maximum heating temperature, and its average temperature was higher (as shown in Table 5). When the temperature is too high, the excessive flow or expansion of asphalt causes the structure of the mixture to change, resulting in a decrease in the healing rate under the controlled temperature of H6.

In addition, in the H1 (unheated) state, it was found that the incorporation of SF weakened the healing rate at room temperature. For samples with high SF content (8% and 10%), the HR_S_ was about 14%. Compared with the control group (sample with 0% SF content), the HR_S_ was 34.96%, which was reduced by 20.96%. This can be attributed to two reasons: On the one hand, due to the reinforcing effect of SF, the sample with more SF content has a higher initial fracture strength. When it breaks, the reinforcing effect of SF is lost, and it shows a similar fracture strength after healing. On the other hand, the SFs limit the diffusion of surrounding asphalt between the two crack fracture surfaces [46].

## 4. Conclusions

This study evaluates the comprehensive effect of SF contents on the road performance, thermal, and healing properties of electromagnetic induction-heated asphalt wearing courses. Based on the above results, the following conclusions are drawn:

1. The addition of SF can improve the high-temperature stability, low-temperature and intermediate-temperature crack resistance, and moisture stability of the asphalt wearing course, but it has an adverse effect on the volume performance and anti-skid performance.
2. Samples with the same SF content exhibit uneven temperature distribution characteristics. The maximum, minimum, and average surface temperatures are all highly linearly related to heating time. The heating temperature increases with SF content, but the higher maximum heating rate causes worse heating uniformity and lowers the healing rate. Among them, the maximum temperature is most affected by the SF content. The maximum heating rate of the sample with 10% SF reached 3.92 °C/s, while its heating rate at the minimum temperature was similar to that of the sample with 6% SF, which was only about 0.7 °C/s, indicating the worst heating uniformity.
3. When heated for 30 to 40 s, samples with 8% and 10% SF will experience local structural damage due to the maximum temperature exceeding 180 °C, while samples with 2%, 4%, and 6% SF content will not show structural damage when heated for 60 s. Therefore, a heating method that controls the maximum temperature of the upper surface is a convenient and reliable way to avoid local overheating.
4. The incorporation of SF increases the initial fracture strength while reducing the natural (unheated) healing rate. The best healing level was the sample with 2% SF when the maximum temperature of the upper surface reaches H5 (160 °C). As the SF content decreases, the healing rate increases; however, the heating time also increases, which is not conducive to practical applications.
5. The final recommended optimal SF content is 6% of the asphalt volume. At this time, the asphalt mixture has appropriate VV, VFA, moisture stability, and anti-skid properties, in addition to the best high-temperature stability, low-temperature crack resistance, and intermediate-temperature crack resistance. Meanwhile, it also exhibits uniform temperature distribution and high healing efficiency.

## Figures and Tables

**Table 1:** Technical information of HVA.

Test Items	Unit	Measured Value	Specified Range	Specification
Penetration (25 °C, 100 g, 5 s)	0.1 mm	45	40–55	T 0604-2011
Softening point	°C	96	≥90	T 0606-2011
Ductility (5 cm/min, 5 °C)	cm	38	≥30	T 0605-2011
Dynamic viscosity (60 °C)	Pa·s	178,468	≥100,000	T 0620-2000
Rotational viscosity at 135 °C	Pa·s	5.19	≤7	T 0625-2011
Rotational viscosity at 175 °C	Pa·s	1.193	≤1.5	T 0625-2011

**Table 2:** Technical information of basalt aggregate.

Test Items	Unit	Measured Value	Specified Range	Specification
Apparent relative density	g/cm^3^	3.021	≥2.7	T 0304-2005
Flakiness and elongation	%	3.7	≤12	T 0312-2005
Los Angeles abrasion	%	7.4	≤20	T 0317-2005
Crushed ratio	%	11.6	≤18	T 0316-2005
Water absorption rate	%	0.81	≤1	T 0304-2005
Ruggedness	%	2.7	≤8	T 0314-2005
Polished stone value	%	49	≥42	T 0321-2005

**Table 3:** Properties of steel fiber.

Parameter	Unit	Measured Value
Density	g/cm^3^	7.8
Oil content	%	<0.2
Equivalent diameter	μm	70–130
Average fiber length	mm	4.2
Melting point	°C	1530

**Table 4:** Linear fitting results of heating time and temperature.

SF Content (%)	Temperature Type	Linear Fitting Formula	R^2^
2	Max	Y = 45.47 + 0.72x	0.99241
	Min	Y = 24.10 + 0.51x	0.98767
	Avg	Y = 26.05 + 0.71x	0.99418
4	Max	Y = 49.19 + 1.02x	0.99236
	Min	Y = 23.51 + 0.85x	0.98685
	Avg	Y = 28.61 + 1.03x	0.99183
6	Max	Y = 54.28 + 1.51x	0.99721
	Min	Y = 27.97 + 0.89x	0.98706
	Avg	Y = 29.97 + 1.40x	0.99684
8	Max	Y = 18.16 + 3.95x	0.9874
	Min	Y = 16.34 + 1.56x	0.9819
	Avg	Y = 21.10 + 2.20x	0.99753
10	Max	Y = 5.46 + 4.86x	0.98276
	Min	Y = 26.07 + 0.91x	0.99633
	Avg	Y = 27.47 + 1.91x	0.99876

**Table 5:** The maximum temperature control matrix of the upper surface during the healing process and its corresponding heating time and average temperature.

SF Content/%	Designation	Maximum Temperature Control Value/°C	Corresponding Average Temperature/°C	Corresponding Heating Time/s	Description
0	H1	-	-		No heating
2	H1	-	-		No heating
	H2	100	79.6	76	
	H3	120	99.4	104	
	H4	140	119.2	132	
	H5	160	138.9	160	
	H6	180	158.7	189	
4	H1	-	-		No heating
	H2	100	79.5	50	
	H3	120	99.8	70	
	H4	140	120.1	89	
	H5	160	140.4	109	
	H6	180	160.8	129	
6	H1	-	-		No heating
	H2	100	72.6	30	
	H3	120	91.4	44	
	H4	140	110.2	57	
	H5	160	129	70	
	H6	180	147.8	83	
8	H1	-	-		No heating
	H2	100	66.5	21	
	H3	120	77.7	26	
	H4	140	88.9	31	
	H5	160	100.1	37	
	H6	180	111.3	42	
10	H1	-	-		No heating
	H2	100	63.9	19	
	H3	120	71.3	24	
	H4	140	78.6	28	
	H5	160	85.9	32	
	H6	180	93.2	36	

## Data Availability

Data are contained within the article.
